# Sexually Transmitted *Neisseria gonorrhoeae* Infections—Update on Drug Treatment and Vaccine Development

**DOI:** 10.3390/medicines8020011

**Published:** 2021-02-05

**Authors:** Amber Jefferson, Amanda Smith, Pius S. Fasinu, Dorothea K. Thompson

**Affiliations:** 1 School of Pharmacy, College of Pharmacy & Health Sciences, Campbell University, Buies Creek, NC 27506, USA; amjefferson0125@email.campbell.edu (A.J.); arsmith0124@email.campbell.edu (A.S.); 2 Department of Pharmaceutical Sciences, College of Pharmacy & Health Sciences, Campbell University, Buies Creek, NC 27506, USA; fasinu@campbell.edu

**Keywords:** gonorrhea, *Neisseria gonorrhoeae*, multidrug resistance, ceftriaxone, azithromycin, proteomics-driven vaccine antigen discovery, in silico approaches to vaccine antigen identification, vaccine development

## Abstract

**Background:** Sexually transmitted gonorrhea, caused by the Gram-negative diplococcus *Neisseria gonorrhoeae*, continues to be a serious global health challenge despite efforts to eradicate it. Multidrug resistance among clinical *N. gonorrhoeae* isolates has limited treatment options, and attempts to develop vaccines have not been successful. **Methods:** A search of published literature was conducted, and information extracted to provide an update on the status of therapeutics and vaccine development for gonorrheal infection. **Results:** Recommended pharmacological treatment for gonorrhea has changed multiple times due to increasing acquisition of resistance to existing antibiotics by *N. gonorrhoeae*. Only broad-spectrum cephalosporin-based combination therapies are currently recommended for treatment of uncomplicated urogenital and anorectal gonococcal infections. With the reported emergence of ceftriaxone resistance, successful strategies addressing the global burden of gonorrhea must include vaccination. Century-old efforts at developing an effective vaccine against gonorrhea, leading to only four clinical trials, have not yielded any successful vaccine. **Conclusions:** While it is important to continue to explore new drugs for the treatment of gonorrhea, the historical trend of resistance acquisition suggests that any long-term strategy should include vaccine development. Advanced technologies in proteomics and in silico approaches to vaccine target identification may provide templates for future success.

## 1. Introduction

Gonorrhea, a sexually transmitted infectious disease caused by the bacterium *Neisseria gonorrhoeae* (gonococcus), has remained an intractable global health problem despite continuing efforts to curtail its health impacts. The public health challenge posed by gonorrhea stems primarily from the large number of asymptomatic infected individuals, the ability of the gonococcus to undergo high levels of surface antigenic variation, and the progressive emergence of antibiotic-resistant gonococcal strains to recommended empiric monotherapies [1]. The World Health Organization (WHO) cited an increase in the number of new and untreated cases globally due to therapeutic failures and the asymptomatic nature of the disease [2]. According to the WHO, approximately 87 million people were diagnosed with gonorrhea out of the 376 million globally reported cases of sexually transmitted infections (STIs) that occurred among 15–49 year olds in 2016. Estimated global prevalence of urogenital gonorrhea was higher among females (0.9%) compared to males (0.7%) [3]. Additionally, the epidemiology of incident cases of gonorrhea varies widely by geographical WHO region, with the highest prevalence in the African region (women 1.9%; men 1.6%), the Americas (women 0.9%; men 0.8%), and the Western Pacific region (women 0.9%; men 0.7%), and the lowest prevalence in Europe (women 0.3%; men 0.3%) [3]. It is believed that these numbers likely underestimate the actual cases of gonorrhea globally [4]. Epidemiological STI surveillance and diagnostics are inadequate in most developing and resource-limited countries, where actual infection numbers are difficult to estimate. Differences in socioeconomic conditions, cultural perceptions of STIs, and access to quality STI education and prevention measures also likely contribute to this heterogeneity in country-specific prevalence data [5]. In 2018, a total of 583,405 gonorrheal cases were reported in the United States alone (i.e., a rate of 179.1 cases per 100,000 population), making gonorrhea the second most commonly reported STI [6]. According to the U.S. Centers for Disease Control and Prevention (CDC), the incidence of gonorrhea continues to rise annually [6]. Case reporting data indicated that the rate of U.S. gonococcal infections increased 5.0% during the period 2017–2018 and increased 82.6% since 2009, when reported gonorrhea cases declined to an historic low [6]. Regional, gender, and ethnicity disparities in rates of reported gonorrhea cases are also observed in the U.S. Surveillance data collected by the CDC revealed that the South had the highest rate of gonorrhea cases in 2018 (194.4 cases per 100,000 population) compared to the Northeast, which had the lowest rate (138.4 cases per 100,000 population) [6]. The rate of reported gonorrhea cases among males increased by a higher amount compared to females (6.0% versus 3.6%, respectively) in 2018. Finally, 2018 rates of reported gonococcal infections were the highest among Blacks (548.9 cases/100,000), American Indians/Alaska Natives (329.5 cases/100,000), Native Hawaiians/Other Pacific Islanders (181.4 cases/100,000), and Hispanics (115.9 cases/100,000), while rates remained the lowest among Whites (71.1 cases/100,000) and Asians (35.1 cases/100,000) [6]. U.S. rates of gonorrhea are also likely underestimated due to incomplete reporting. Furthermore, the majority of infected people are asymptomatic and constitute an important source of infection transmission [1,7]. 

*N. gonorrhoeae* is a human-restricted pathogen that infects the lower genital tract, pharynx, and rectum, with varying degrees of complication depending on the sex of the patient. The predominant site of infection is the cervix in females and the anterior urethra in males. Although gonorrhea is most commonly seen in young individuals 15–24 years of age, it can be present in any sexually active individual [6]. Symptoms of gonorrhea include cervical or urethral purulent discharge, discomfort, dysuria, urethritis, or cervicitis. Untreated cervical infection may result in ascension of the gonococcus to the upper genital tract and lead to serious reproductive health complications such as pelvic inflammatory disease (PID), chronic pelvic pain, ectopic pregnancy, and tubal infertility [7,8]. Vertical transmission is a significant concern for pregnant women infected with *N. gonorrhoeae* and may lead to chorioamnionitis, septic abortion, premature rupture, preterm delivery, and sight-threatening neonatal conjunctivitis [9]. In males, rare disease complications and sequelae of untreated urethritis include penile edema, urethral stricture, epididymitis, or prostatitis [10]. Untreated urogenital gonorrhea infrequently disseminates to extragenital anatomic sites, causing septic arthritis, endocarditis, and skin manifestations in both genders. Moreover, *N. gonorrhoeae* infection facilitates the acquisition and transmission of other STIs, most notably HIV infection [11].

The lack of natural immunity in both symptomatic and asymptomatic patients has impeded development of an effective anti-gonococcal vaccine. Strain-specific antibodies directed against a number of gonococcal antigens (e.g., lipooligosaccharide [LOS], pili, and outer membrane proteins [PorB, Opa]) have been detected in the sera, seminal plasma, and cervical secretions of individuals infected with *N. gonorrhoeae* (reviewed in [7]). However, these humoral immune responses to *N. gonorrhoeae* tend to be modest, with antibody production in infected individuals characterized as only slightly increased compared to uninfected individuals and not protective against reinfection [12]. While in vitro evidence exists for some anti-gonococcal antibodies promoting either complement-mediated killing or opsonin-facilitated phagocytosis of the gonococcus [13,14], the potential role of these antibodies in immunologic clearance of the pathogen from the host remains unclear. The persistence of gonococcal infections and the common occurrence of reinfection are consistent with evidence demonstrating that *N. gonorrhoeae* is capable of evading and actively suppressing the immune response to promote its survival in the host (reviewed in [15]). These evasion and suppression mechanisms include sialylation of the gonococcal LOS [16,17] and induction of anti-gonococcal Rmp (reduction-modifiable protein) antibodies that block the bactericidal activity of IgG antibodies directed against gonococcal LOS or porin protein [18]. In addition to non-protective humoral immune responses, *N. gonorrhoeae* infection manipulates the adaptive cell-mediated response away from Th1/Th2-based immunity and towards a Th17-driven neutrophilic inflammatory response by inducing localized TGF-β and IL-10 cytokine production [19,20]. This immunologic bias toward neutrophilic influx results in the cellular damage associated with serious clinical sequelae of gonorrhea and prevents the development of protective immunity ([21]; reviewed recently in [8]).

In the absence of an effective vaccine and protective natural immunity against repeated infection, public health control of gonorrhea has depended exclusively upon effective and affordable antibiotic pharmacotherapy [15]. However, the continued resistance of gonococcal strains to all antimicrobial drugs introduced as first-line treatment since the 1930s points to the possibility of widespread, untreatable *N. gonorrhoeae* infections in the future. With *N. gonorrhoeae* assigned to ‘superbug’ status by the WHO, it is imperative that novel antibiotics are developed and new potential targets for vaccine design are identified in order to address the urgent need for prevention and treatment of gonorrhea. While a number of reviews have been published on gonorrhea over the years, including some that are recent [1,5,15], there is a current need for a scholarly focus on potential novel therapeutics for the treatment of *N. gonorrhoeae* infection and on new approaches to antigen target discovery for vaccine development. The aim of the current study, therefore, is to review the evolution of chemotherapeutic treatment options for gonorrhea, discuss the treatment potential of new therapeutics, and provide an update on the progress toward the development of an anti-gonococcal vaccine, with a concentration on recent discoveries related to vaccine antigen identification.

## 2. Methods 

A literature search was conducted in November 2019 and updated in November 2020 using the PubMed, Scopus, clinicaltrials.gov, and Google Scholar databases. All available publications associated with anti-gonococcal therapeutics and vaccine development were included. The literature search was conducted using the following search terms and combinations thereof: “*Neisseria gonorrhoeae*”, “gonorrhea”, “drug treatments”, “vaccine”, “vaccine development”, “novel drug or vaccine”, “future treatments”, “meningococcal vaccine”, and “vaccine clinical trials”. All full-text articles including randomized controlled trials (RCTs), meta-analyses, clinical trials, and systematic reviews were screened for inclusion in this extensive review. Only primary studies conducted on new therapeutics and vaccine development for gonorrhea were included for extensive review. Articles were excluded if they were published in languages other than English. The date of publication was not restricted in our screening process. To circumvent bias, two reviewers independently searched the literature and assessed the eligibility of each study for inclusion based on the predefined criteria. Two additional reviewers oversaw the study selection process. 

## 3. Results

**Figure 1:**
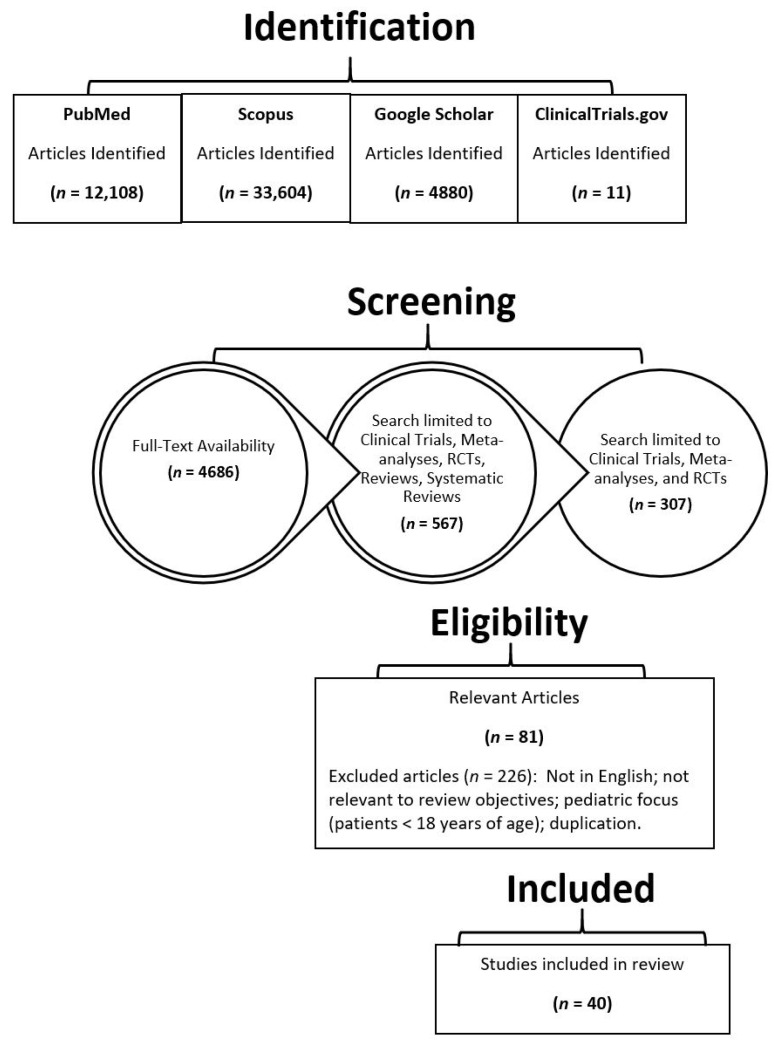
Search results and study selection.

Figure 1 summarizes the study selection process and the search results obtained. The initial search yielded articles in excess of 50,000. After screening and excluding duplications, 40 articles were identified for extensive review.

### 3.1. Historical and Current Antibiotic Regimens for Treating Gonorrhea

**Figure 2:**
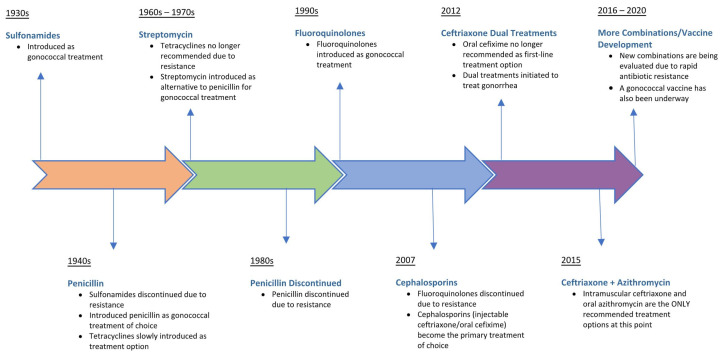
Evolution of recommended drug treatments for *Neisseria gonorrhoeae* infections. Treatment introduced (pill symbol). Treatment discontinued (circle-backslash symbol).

As a sexually transmitted infection with centuries of history and a human-specific pathology, *N. gonorrhoeae* has been exposed to many of the existing antibiotics in the antimicrobial armamentarium, with the consequent development of resistance to a wide range of chemotherapeutic drugs. Figure 2 illustrates the evolution of first-line antibiotic regimens for treating uncomplicated gonorrhea since the introduction of sulfonamides in the mid-1930s. Temporal shifts in the epidemiology of antimicrobial resistance patterns for *N. gonorrhoeae* have significantly impacted treatment recommendations. Gonococcal strains have emerged worldwide that are resistant to all previous empirical monotherapies, including sulfonamides, penicillins, earlier-generation cephalosporins, tetracyclines, streptomycin, and fluoroquinolones [1,6,22]. Resistance to sulfonamides, the first treatment regimen for gonorrhea in the antibiotic pipeline, developed due to mutations acquired in the folP gene, which reduced the binding affinity of sulfonamide for its target, dihydropteroate synthase [1]. Introduced into clinical practice in the period 1943–1944, penicillin was used to effectively treat gonorrhea for decades until the emergence of resistant strains possessing a plasmid-mediated β-lactamase, an enzyme that inactivates β-lactam antibiotics. Alternative antibiotics such as tetracycline were also effective in treating gonorrhea until the occurrence of resistant *N. gonorrhoeae* strains harboring the ribosomal protection protein TetM which led to the discontinuation of tetracycline as a treatment option [1]. Overexpression of efflux pumps, reduced uptake, and modification of drug targets have been identified as other mechanisms by which *N. gonorrhoeae* has developed resistance to antibiotics in the treatment pipeline [23].

Following the introduction of fluoroquinolones as anti-gonococcal therapy, ciprofloxacin-resistant *N. gonorrhoeae* strains emerged as a result of selection of genetic mutations in gyrA and parC, which reduced drug binding to targets DNA gyrase and topoisomerase IV, respectively [1]. Not surprisingly, fluoroquinolones were removed from the CDC-recommended guidelines in 2007 (Figure 2), leaving extended-spectrum cephalosporins as the last bulwark in empiric anti-gonococcal monotherapy. Third-generation cephalosporins (oral cefixime and injectable ceftriaxone) were the treatment of choice until 2012 when a rising rate of gonococcal resistance prompted the CDC to recommend removing cefixime from treatment regimens [6]. Emerging resistance and clinical treatment failures associated with ceftriaxone use have already been documented in countries outside the US, highlighting the possible transmission of untreatable gonorrhea [24,25]. As of 2020, the CDC guidelines recommend combination antibiotic therapy consisting of a single intramuscular dose of ceftriaxone (250 mg) and a single oral dose of macrolide azithromycin (1 g) administered simultaneously as the first-line empirical treatment for uncomplicated urogenital and anorectal gonococcal infections [6]. Of serious concern, a gonococcal strain resistant to the currently recommended dual antimicrobial therapy was recently isolated from a patient with pharyngeal gonorrhea, resulting in the first recorded clinical failure of ceftriaxone and azithromycin [26]. Since then, clinical isolates of *N. gonorrhoeae* exhibiting combined high-level resistance to azithromycin and resistance to ceftriaxone have been documented as part of public health surveillance programs in the United Kingdom and Australia [27]. It is therefore unlikely that dual antimicrobial regimens will completely prevent resistance emergence among *N. gonorrhoeae* strains, requiring the development of novel, affordable antibiotics for monotherapies or inclusion in new dual treatment regimens [23]. The acquisition and spread of antimicrobial resistance determinants among naturally competent gonococcal strains means that the antibiotic pipeline will need to be replenished with new, efficacious drug therapies in order to meet the therapeutic challenge of treating gonorrhea. However, bacterial drug targets are finite in number, and preventative vaccination is the optimal long-term approach to controlling gonococcal infection and reducing disease burden.

### 3.2. Vaccine Development: Clinical Trials, Human Challenge Trials, and Observational Human Studies

Recorded efforts to develop a vaccine to protect against *N. gonorrhoeae* infection date back to the early 1900s. Since that time, only four candidate gonococcal vaccines have advanced to the stage of clinical trials or human challenge trials. Little information is available on the first, now century-old, clinical evaluation of a gonococcal whole-cell vaccine [28]. The second was a parenterally administered crude, heat-killed, partially lysed whole-cell vaccine, which was evaluated via a double-blind placebo-controlled study conducted in a population of 62 Inuit volunteers in northern Canada [29]. There was a high incidence and prevalence of *N. gonorrhoeae* in this study population. Of the 33 subjects vaccinated and the 24 subjects in the placebo group, 10 (30%) and 7 (24%), respectively, contracted gonorrhea in the year following vaccination, resulting in no significant difference between the control and experimental groups (*p* = 0.78) [29]. The third candidate vaccine consisted of a single-antigen purified pilus that was examined in a double-blind placebo-controlled trial involving the randomization of 3250 US military personnel in Korea [30]. The gonococcal pilus vaccine was delivered intradermally to volunteers using a two-dose regimen separated by 14 days. The vaccine failed to protect male volunteers against gonococcal urethritis, with 108 and 102 subjects from the vaccine and placebo groups, respectively, acquiring gonorrhea 15 days or more after inoculation [30]. Both the killed whole-cell and purified pilin vaccines failed to induce protection against heterologous reinfection. Finally, a human vaccine challenge study involved parenterally immunizing male subjects with purified gonococcal protein I, the major outer membrane porin protein that constitutes a channel for the entry of small molecules through the outer membrane of *N. gonorrhoeae*. The protein I-based vaccine elicited a significant antibody response in subjects (similar to the pilus-based vaccine), but offered no protection against an intraurethral challenge in men with the homologous gonococcal strain (reviewed in [31]). Beyond these four candidate vaccines, no preclinical development of gonococcal vaccine targets has progressed to human studies, underscoring the challenges and difficulties associated with *N. gonorrhoeae* vaccine development. The primary obstacles include the lack of known correlates of immune protection against natural mucosal infections and the high degree of phase and antigenic variability of *N. gonorrhoeae* surface antigens (reviewed in [1]). The failure of early clinical trials and vaccine studies was likely due, in part, to the fact that antigenically variable gonococcal pilin and porin PorB proteins were targeted, resulting in a lack of broadly protective antibody responses. 

Recently, novel high-throughput approaches, such as proteomics-driven reverse vaccinology and in silico subtractive genomics (discussed in more detail in the following sections), are being applied to the task of identifying new surface-expressed and antigenically conserved proteins in *N. gonorrhoeae* that could be explored as targets for vaccine development. Other promising preclinical efforts have focused on the design of novel vaccine platforms, antigen delivery systems and adjuvants, including bacterial ‘ghosts’ (empty bacterial cell envelopes) as potent carriers for the delivery of a PorB-based DNA vaccine [32] and microneedle skin patches for transdermal delivery of formalin-fixed *N. gonorrhoeae* whole cells [33]. In a study reported by Jiao et al. [32], a novel *N. gonorrhoeae* DNA vaccine was constructed by inserting the full-length porB gene into the eukaryotic expression vector pVAX1 and then the recombinant pVAX1-porB plasmid was loaded into *Salmonella enteritidis* (SE) ghosts for delivery. Mice orally immunized with SE ghosts (+ pVAX1-porB) elicited gonococcal PorB-specific serum antibodies that had bactericidal activity in contrast to plasmid DNA alone, suggesting that the SE ghosts had a strong adjuvant effect on the immunogenicity of the DNA vaccine. Moreover, SE ghosts (+ pVAX1-porB) promoted dendritic cell maturation and induced a strong cellular immune response in immunized mice, with increased levels of CD3^+^, CD4^+^, and CD8^+^ T cell subpopulations [32]. While PorB-based DNA vaccines have not progressed beyond the preclinical evaluation stage, the bacterial ‘ghost’ platform technology does offer a potential approach to enhancing the immunogenicity of a *N. gonorrhoeae* vaccine target. 

The recent development of an effective vaccine against the closely related *Neisseria meningitidis* serogroup B, combined with new tools for vaccine antigen mining, has raised optimism that an efficacious *N. gonorrhoeae* vaccine may be possible in the near future. While the infectious pathologies of these strictly human pathogens are distinct, *N. gonorrhoeae* and *N. meningitidis* exhibit 80–90% genomic identity at the nucleotide sequence level, with many surface-exposed proteins sharing a high degree of sequence identity (e.g., methionine sulfoxide reductase MsrA/B shares 98% amino acid identity) [34,35]. Studies employing epidemiological surveillance data suggest that outer membrane vesicle (OMV) *N. meningitidis* group B vaccines reduce the incidence of gonorrhea (Table 1; [36,37]). In a multiclinic retrospective case–control study, Petousis-Harris et al. [36] investigated the association of immunization with a meningococcal group B OMV vaccine (trade name MeNZB) and gonorrhea diagnosis. Vaccinated subjects were those individuals who had received the serogroup B OMV vaccine MeNZB as part of a national immunization campaign in New Zealand. Study results showed that MeNZB-immunized subjects were significantly less likely to be diagnosed with gonorrhea over a 10 year period following vaccination than unimmunized subjects [36]. After adjusting for potential confounding factors, the effectiveness of the MeNZB vaccine against gonorrhea was estimated to be 31%. While the estimated vaccine efficacy was modest, this was the first report of a vaccine providing some degree of protective immunity against gonorrhea in humans. Similarly, another epidemiological study observed decreased rates of gonorrhea incidence in the general public following mass vaccination with the meningococcal serogroup B vaccine VA-MENGOC-BC in Cuba [37]. A recent investigation expanded on the New Zealand findings by demonstrating that individuals immunized with the new four-component meningococcal group B vaccine 4CMenB, trade name Bexsero^®^ (GlaxoSmithKline), induced antibodies in humans that specifically recognized gonococcal proteins [38]. Bexsero^®^ contains the antigen components of MeNZB (meningococcal group B OMVs) in addition to three recombinant meningococcal antigens (*Neisseria* adhesin A, NadA; factor H-binding protein, fHbp; Neisserial heparin-binding antigen, NHBA) (Table 1). Semchenko et al. [38] demonstrated that anti-gonococcal antibodies generated as a result of Bexsero^®^ immunization cross-reacted with the highly conserved NHBA protein in *N. gonorrhoeae*, suggesting that Bexsero^®^ may provide additional protection above what has been observed for MeNZB. Collectively, these studies suggest the potential utility of OMV meningococcal serogroup B vaccines in eliciting a cross-protective effect against *N. gonorrhoeae* and may help to reduce the global incidence of gonorrhea in the absence of a specific effective vaccine.

### 3.3. Current Status of Neisseria gonorrhoeae Antigens for Vaccine Development

Safe and effective vaccines against gonococcal infections are desperately needed to contain the global emergence of multidrug-resistant *N. gonorrhoeae* strains. With currently no new antibiotics for monotherapy in the drug pipeline, health care practitioners are left to administer aggressive and invasive dual therapy regimens to treat gonorrhea. However, the ability of gonococci to rapidly acquire drug resistance suggests that even newly developed, novel antibiotics would eventually become ineffective. Development of an effective anti-gonococcal vaccine is, therefore, the only sustainable approach to controlling the proliferation of *N. gonorrhoeae* infections [39]. The identification of vaccine antigens is critical to this endeavor, and anti-gonococcal vaccine development continues to remain largely in the antigen discovery phase. Ideally, such vaccine antigens should be highly conserved and broadly distributed among diverse gonococcal strains, immunogenic, and capable of eliciting bactericidal or opsonic antibodies or antibodies that block essential physiological functions of *N. gonorrhoeae*. In contrast to the closely related *Neisseria meningitidis*, *N. gonorrhoeae* does not express a surface capsule, which constitutes a potent immunogenic target in several FDA-approved meningococcal vaccines (e.g., Menomune, Menactra, and Menveo). As a result, gonococcal vaccine research has focused on proteinaceous targets such as outer membrane proteins and surface-accessible lipoproteins.

As presented in Table 1, numerous gonococcal surface-exposed structures capable of inducing bactericidal antibodies in immunized animals have been identified and proposed as potential vaccine candidates. The vast majority of these vaccine targets play critical roles in various physiologic functions of the pathogen and include adherence and invasion of mucosal epithelial cells [40,41,42,43,44,45], iron acquisition [46], immune evasion due to serum complement resistance [47,48] and efflux of neutrophil-derived antimicrobial peptides [49,50], cellular metabolism [51], and oxidative stress protection [35]. By contrast, one novel study focused on the presence of filamentous NgoΦfil bacteriophage (phage) DNA in gonococcal genomes as a potential source for vaccine candidates (Table 1). Phage proteins displayed on the surface of *N. gonorrhoeae* cells were shown to elicit bactericidal antibodies in rabbits, and anti-NgoΦfil protein antibodies blocked gonococcal adherence to human endocervical cells [52].

Gonococcal adhesion to human mucosal epithelium constitutes the primary event in the establishment of *N. gonorrhoeae* infection (reviewed in [53]), and consequently, vaccine development efforts have concentrated on the utility of Type IV pili, opacity (Opa) proteins, and major porin protein PorB as vaccine targets (Table 1). Initial attachment of *N. gonorrhoeae* to the epithelial cell surface and subsequent colonization is mediated largely by Type IV pili. Adhesins such as the outer membrane Opa proteins facilitate more intimate interactions of *N. gonorrhoeae* with carcinoembryonic antigen-related cell adhesion molecule (CEACAM) receptors on epithelial cells [41,54]. Type IV pili and Opa proteins are expressed during natural infection and are essential factors in the ability of gonococci to colonize the genital tract epithelium (reviewed in [53]). PorB is the most abundant protein in the gonococcal outer membrane and influences bacterial colonization and invasion, as well as contributing to serum complement resistance ([42]; reviewed in [15]). At first glance, these gonococcal antigens—pilin, Opa, and PorB—appear to be ideal targets for a vaccine because of their surface accessibility and essential roles in the critical first step in *N. gonorrhoeae* pathogenesis. These gonococcal surface antigens, however, are subjected to genetic inter- and intravariability. In the case of Type IV pili, for instance, intrachromosomal recombination events at the expression locus of the major pilus subunit create antigenically distinct pilin variants (reviewed in [15]). Additionally, *opa* genes undergo phase variation in which their expression in the gonococcus is reversibly turned on or off, making these surface antigens challenging as vaccine targets. Targeting the relatively conserved semi-variable (SV) loop of mature Opa proteins with cyclic peptides, however, has shown some potential in stimulating a humoral response that recognizes a wide range of antigenically distinct Opa variants [44]. 

Other gonococcal antigens have been identified as inducing a functional antibody response in laboratory animals and are characterized by antigenic conservation and stable expression between *N. gonorrhoeae* strains and within the same strain, attracting research interest in their possible utility as vaccine targets. These potential vaccine candidates most notably include transferrin-binding proteins A and B [46]; MtrE of the MtrCDE multidrug transporter system [49,50]; nitrite reductase AniA [51]; methionine sulfoxide reductase MsrA/B [35]; and neisserial surface protein A (NspA) [47,48] (Table 1). MtrE and NspA are stably expressed proteins with surface-exposed epitopes that are highly conserved at the sequence level among gonococcal strains; both proteins contribute to the ability of *N. gonorrhoeae* to evade host innate immune defenses [48,49]. In addition, MtrCDE was previously shown to be essential for the in vivo biological fitness of *N. gonorrhoeae* in a murine vaginal tract infection model [55,56] and is upregulated in antibiotic-resistant *N. gonorrhoeae* strains, resulting in elevated minimal inhibitory concentrations (MICs) of antibiotics [57]. Studies demonstrated that mice immunized with the surface-expressed Loop 2 of MtrE and a recombinant plasmid encoding gonococcal NspA generated target-specific antibody titers that displayed bactericidal activity [47,50]. Both MtrE and NspA meet the criteria for a successful vaccine antigen. 

In the absence of an effective FDA-approved prophylactic against *N. gonorrhoeae* infections, new strategies are needed to expand the pool of candidates considered for potential clinical vaccine development. One innovative approach is reverse vaccinology antigen mining using a combination of high-throughput quantitative proteomics and bioinformatics (recently reviewed in [58]). The utility of proteomic technology has been clearly demonstrated in the discovery of *N. gonorrhoeae* lipoprotein MetQ (NGO2139). Initially, this functionally uncharacterized protein (NGO2139) was identified as a result of high-throughput proteomic profiling of *N. gonorrhoeae* strain FA1090 grown under three environmental conditions mimicking in vivo microecological niches in the human host: (i) exposure to normal human serum, (ii) iron limitation, and (iii) oxygen deprivation (i.e., anaerobiosis) [59]. NGO2139 was discovered as part of a proteome subset of ubiquitous proteins whose expression remained unchanged upon exposure of *N. gonorrhoeae* to normal human serum, iron depletion, and anaerobic conditions. Rabbit anti-NGO2139 antibodies exhibited bactericidal activity and cross-reacted against diverse gonococcal isolates [59]. Bioinformatics revealed that NGO2139 contains a classical lipoprotein signal peptide indicative of outer membrane-localized proteins [59]. A subsequent investigation verified the functional role of NGO2139 as the L-methionine-binding component, MetQ, of an ABC transporter system and showed that MetQ is involved in gonococcal adherence to cervical epithelial cells [60]. Semchenko and colleagues [60] demonstrated that mouse anti-MetQ polyclonal antibodies were not only bactericidal, but also reduced adherence of *N. gonorrhoeae* to cervical epithelial cells. Furthermore, MetQ is widely distributed. Homologs of this proteomics-derived vaccine candidate are present in all 14 *N. gonorrhoeae* reference strains comprising the 2016 WHO panel, as well as in 3 examined *Neisseria meningitidis* strains [39]. Most recently, a preclinical assessment of MetQ was conducted using a female mouse model of genital tract infection. Sikora et al. [61] created a recombinant protein construct with the most phylogenetically prevalent allele of MetQ (rMetQ) and formulated rMetQ with the adjuvant CpG. Mice were immunized with rMetQ-CpG by subcutaneous injection, followed by three intranasal boosts, and subsequently challenged with *N. gonorrhoeae*. Mice immunized with rMetQ-CpG cleared the experimental infection significantly faster than control mice, prompting researchers to propose the inclusion of MetQ in a broad-spectrum subunit vaccine for gonorrhea [61]. Other proteomics-derived vaccine candidates should be prioritized for preclinical evaluation based on the criteria of conservation, function in *N. gonorrhoeae* pathogenesis, and immunogenicity.

**Table 1:** Potential Antigen Targets for Gonorrhea Vaccine and Drug Development.

Target	Study Design	Functional Role	Immunogenic Effect or Drug Binding Potential	Candidate Antigen Attributes	References
**Adherence and Invasion of Mucosal Epithelial Cells**					
Porin (PorB)	In vitro cell-based studies	Outer membrane protein Contributes to host cell invasion and serum complement resistance	Ability to form self-aggregating micellular structures (adjuvant effect); antibodies not cross-protective against heterologous strains	Antigenically variable surface-exposed sequences	[42,43,45]
Type IV pilin	In vitro cell-based studies, immunization	Outer membrane protein Mediates adherence to epithelial cells	Antibodies generated against synthetic peptides representing gonococcal pilin sequences blocked GC adhesion	Phase and antigenically variable	[40]
Opacity associated (Opa) protein	In vitro cell-based studies, immunization	Outer membrane protein Mediates adherence to human carcinoembryonic antigen-related cellular adhesion molecules (CEACAMs) and invasion of host cells	Bactericidal antibodies generated to linear hypervariable (HV_2_) Opa loop peptides	Phase variable	[41,44,54]
Interaction of pilin-linked glycan with terminal galactose	Peptide library screening, in vitro cell-based studies	Critical for initial interaction of GC with cervical epithelial cells via galactose-binding I-domain of host complement receptor 3 (CR3)	Methyldopa and carbamazepine blocked pilin glycan–CR3 I-domain interaction (host-targeted therapy)	Increased efficacy compared to ceftriaxone in curing cervical infection ex vivo	[62]
**Iron Acquisition**					
TbpA/B	Genetic chimera approach, mice	Transferrin-binding protein A Transferrin-binding protein B	Elicited bactericidal Tbp-specific antibodies against homologous and heterologous strains	Conserved Tbp epitopes	[46]
**Immune Evasion and Virulence**					
MtrE	In vitro cell-based studies	Outer membrane channel of the multiple transferable resistance (Mtr) system (MtrCDE) Contributes to survival of GC in the presence of human neutrophils and neutrophil-derived antimicrobial peptides	Elicited bactericidal antibodies	Highly conserved, surface exposed	[49,50]
NspA	In vitro cell-based studies, immunization	Neisserial surface protein A Binds complement inhibitor Factor H Contributes to gonococcal complement resistance	Elicited NspA-specific IgG and IgA antibodies with bactericidal and opsonic activities	Highly conserved, surface exposed	[47,48]
Peptide mimic of glycan epitope (2C7) of GC LOS	Mouse model, in vitro cell-based studies	Common oligosaccharide structure in LOS Increases survival of GC in humans and mouse model Mice immunized with multi-antigenic peptide mimic of 2C7 epitope (MAP1)	Elicited Th1-biased and bactericidal anti-LOS IgG antibodies	Conserved	[63,64,65]
Peptide mimic of glycan epitope (2C7) of GC LOS	Preclinical experimental infection model	Peptide mimic of 2C7 epitope Configured as a tetrapeptide with glucopyranosyl lipid A in an oil-in-water nanoemulsion (candidate gonococcal peptide vaccine TMCP2)	Elicited bactericidal antibodies and reduced duration/burden of gonococcal cervicovaginal colonization in mice	Conserved, immunogenic	[66]
**Vaccine/Drug Antigens Identified by In Silico and Proteomic Approaches**					
YP_208704.1 (DapD)	Codon biasing, subtractive genomics	2,3,4,5-Tetrahydropyridine-2,6-Dicarboxylate N-succinyltransferase Involved in L-Lysine biosynthesis	Docking study indicated binding of ligand/drug molecule ZINC06311339 (C18H22N6S3) to active site	Essential, cytosolic	[67]
NGO0690	Reverse vaccinology, bioinformatics	Hypothetical P/OM lipoprotein Possible role in threonine biosynthesis and pilin antigenicity Present in all gonococcal strains examined and part of the gonococcal core genome	Anti-NGO0690 antibodies bactericidal against all 4 diverse gonococcal strains tested (low bactericidal titers)	Largely conserved with a small range of allelic variability	[68]
NGO0948	Reverse vaccinology, bioinformatics	P/OM lipoprotein of the NlpB family Homology to BamC of the Bam complex involved in membrane biogenesis Present in all gonococcal strains examined and part of the gonococcal core genome	Anti-NGO0948 antibodies bactericidal against all 4 diverse gonococcal strains tested (low bactericidal titers)	Largely conserved with a small range of allelic variability	[68]
NGO1043	Reverse vaccinology, bioinformatics	Hypothetical P/OM lipoprotein Homology to meningococcal antigen Ag473 Present in all gonococcal strains examined and part of the gonococcal core genome	Anti-NGO1043 antibodies exhibited low-titered bactericidal activity against 3/4 diverse gonococcal strains tested	Largely conserved with a small range of allelic variability	[68]
NGO1701	Reverse vaccinology, bioinformatics	Hypothetical periplasmic protein Homology to copper-binding proteins Present in all gonococcal strains examined and part of the gonococcal core genome	Anti-NGO1701 antibodies bactericidal against all 4 diverse gonococcal strains tested (low bactericidal titers)	Largely conserved with a small range of allelic variability	[68]
LptD (NGO1715)	Proteomics-driven reverse vaccinology	Involved in LOS assembly	Elicited bactericidal antibodies	Highly conserved, surface exposed	[59]
BamA (NGO1801)	Proteomics-driven reverse vaccinology	Involved in binding and inserting beta-barrel proteins into the OM	Elicited bactericidal antibodies	Highly conserved, surface exposed	[59]
TamA (NGO1956)	Proteomics-driven reverse vaccinology	Involved in translocation assembly	Elicited bactericidal antibodies	Highly conserved, surface exposed	[59]
MetQ (NGO2139)	Proteomics-driven reverse vaccinology, bioinformatics, mouse model	L-Methionine-binding lipoprotein Involved in methionine transport	Elicited bactericidal antibodies MetQ formulated with CpG (rMetQ-CpG) accelerated GC clearance from challenged mice and induced a protective immune response in mice	Highly conserved, surface exposed	[39,59,61]
NGO2054	Proteomics-driven reverse vaccinology	Hypothetical protein Predicted signal peptide suggests OM localization	Elicited bactericidal antibodies	Highly conserved, surface exposed	[59]
NGO0282 NGO0439 NGO1688 NGO1889 NGO2105	Quantitative proteomic profiling of WHO reference strains ^†^ and FA6140	LPS/LOS assembly lipoprotein (NGO0282, LptE) OM lipoprotein (NGO0439, LolB) OM protein (NGO1688, OmpU) Lipoprotein (NGO1889, Lpr) Adhesion and penetration protein (NGO2105, AidA)	ND	Conserved, localized to cell envelope or outer membrane fraction	[39]
**Metabolism and Oxidative Stress Protection**					
AniA	Mice, in vitro cell-based studies	Copper-containing OM nitrite reductase Catalyzes the reduction of nitrite to nitric oxide Essential for growth and survival of GC under oxygen-limiting conditions	Antibodies block nitrite reductase function in a whole-cell assay	Conserved, surface exposed	[51]
MsrA/B	Mice, in vitro cell-based studies	Methionine sulfoxide reductase (MsrA/B) Reduces methionine sulfoxide to methionine in oxidized proteins Protects GC against oxidative stress	Anti-MsrA/B antibodies mediate bactericidal and opsonophagocytic killing of GC; capable of functional blocking of MsrA/B activity	Highly conserved, surface exposed	[35]
**Lysogenic Phage**					
Filamentous bacteriophage (NgoΦfil) proteins	Rabbits, in vitro cell-based studies	NgoΦ6 filamentous phage genes in GC Encode proteins needed for progeny phage production	Anti-phage IgG and IgA bound to surface of GC cells; sera exhibited bactericidal activity and blocked GC adherence to cervical epithelial cells	Phage genes present in all sequenced GC strains; phage proteins are surface exposed	[52]
**Inactivated Whole Cells**					
Inactivated whole-cell *N. gonorrhoeae* strain CDC-F62	Nanotechnology, mouse model, in vitro cell-based studies	Whole-cell formalin-fixed GC Encapsulated in biodegradable, albumin-based microparticles for sustained slow antigen release Loaded into microneedle skin patch for transdermal vaccine delivery	Induced antigen-specific IgG and antigen-specific adaptive cellular immunity (CD4 and CD8 lymphocytes) in mice	Immunogenic epitopes preserved	[33]
**Meningococcal Vaccines**					
Meningococcal serogroup B outer membrane vesicle (OMV) vaccine MeNZB	Retrospective case–control study	MeNZB vaccine Used to control meningococcal epidemic in New Zealand >1 million people vaccinated between 2004 and 2008	Associated with decreased rates of gonorrhea; vaccinated individuals were less likely to contract gonorrhea than unvaccinated controls	High genome identity between MEN and GC	[36]
Meningococcal serogroup B OMV	Observational; analysis of public health statistics (1970–2017)	VA-MENGOC-BC vaccine Used to control meningococcal epidemic in Cuba	VA-MENGOC-BC could induce moderate cross-protection against *N. gonorrhoeae* infection	High genome identity between MEN and GC	[37]
MeNZB OMV + 3 recombinant antigens (NadA, fHbp, NHBA)	Bioinformatics, polyclonal rabbit sera, human sera	Antigen composition of the broad-spectrum serogroup B Meningococcal vaccine Bexsero	Anti-gonococcal antibodies induced by MeNZB OMV; strong immune reactivity of anti-NHBA antibodies to GC	Diverse GC strains contain NHBA homolog; surface exposed	[38]

^†^ 2016 World Health Organization (WHO) reference panel consists of 14 diverse *Neisseria gonorrhoeae* strains. Abbreviations: LOS = lipooligosaccharide; GC = gonococcus (*Neisseria gonorrhoeae*); MEN = meningococcus (*Neisseria meningitidis*); P/OM = periplasmic/outer membrane; NadA = *Neisseria* adhesin A protein; fHbp = factor H-binding protein; NHBA = Neisserial heparin-binding antigen; ND = not determined.

### 3.4. Repurposed Drugs, Novel Drug Targets for Gonorrhea Treatment, and Nanomaterials as Drug Adjuvants

In light of the rapidly dwindling antibiotic options for multidrug-resistant *N. gonorrhoeae* infections, novel therapeutics or repurposed indications for existing drugs used for other diseases are needed for clinical intervention. Repurposing existing drugs has the distinct benefit of more rapidly achieving a solution to the clinical intervention challenge. As discussed earlier in this review, Type IV pili are critical for the adherence of *N. gonorrhoeae* to epithelial cells. Previous studies have shown that gonococci initially interact with complement receptor 3 (CR3) on the surface of the human cervical epithelium via pilin-linked glycan with a terminal galactose [69,70]. The I-domain of CR3 binds the terminal galactose of pilin glycan with high affinity [62,69]. Poole et al. [62] recently mapped the galactose-binding epitope of the CR3 I-domain and showed that a peptide mimic of this region competitively inhibited interaction between *N. gonorrhoeae* and CR3. Screening of a compound library identified two drugs, carbamazepine and methyldopa, currently approved for other clinical indications that were able to bind to the I-domain and block pilin-CR3 interaction (Table 1; [62]). Both drugs, which are orally bioavailable, were found to be more effective than ceftriaxone in curing an infection of primary human cervical cells caused by multidrug-resistant gonococcal strains. Carbamazepine is a drug currently used in the context of epilepsy and nerve pain, while methyldopa is an antihypertensive drug of the alpha-2-agonist class. Poole and colleagues [62] found that the killing of gonococci after a 24 h period of treatment with carbamazepine (≥10 μM) or methyldopa (≥10 μM) was host cell-dependent, as these drugs did not affect gonococcal viability in the absence of cervical epithelial cells. In addition, curing of the cervical infection ex vivo occurred at dose amounts of carbamazepine and methyldopa that were lower than what is currently approved for their respective indications [62]. In the absence of an effective vaccine, potential host-targeted therapies, such as the one described by Poole et al. [62], will need to be explored as alternative treatment modalities for multidrug-resistant gonorrhea. Such host-targeted therapeutics are likely to be more recalcitrant to the development of pathogen resistance.

The process of vaccine antigen identification has been revolutionized by high-throughput, systems-based technologies combined with advanced computational tools. Bioinformatics and omics technologies have expedited the target discovery phase of drug development by eliminating time-consuming trial-and-error experimentation and prioritizing the theoretically most promising candidate drug targets for in-depth laboratory testing. One such omics-based approach is subtractive genomics in which species-specific novel therapeutic targets are identified in silico by subtracting strain-specific paralogous and host-specific homologous sequences from the bacterial proteome, leaving pathogen-specific core genes essential to such crucial cellular functions as energy metabolism and amino acid biosynthesis. This approach has been used to identify potential drug and vaccine targets in *N. gonorrhoeae* [67,71]. Most recently, Tanwer et al. [67] employed in silico subtractive genomics, combined with molecular docking analyses, to identify 29 proteins essential to gonococcal survival and propagation, including a cytosolic succinyltransferase (DapD) with a predicted function in L-lysine biosynthesis (Table 1). Codon adaptation indexes (CAIs) were calculated for neisserial gene sequences to predict essential genes in the *N. gonorrhoeae* genome (sequences with a CAI > 0.75 were further examined). Applying molecular docking analyses, a novel lead compound, ZINC06311339 (C18H22N6S3), was predicted to bind to the active site of DapD through simulated protein-ligand interaction [67]. Although experimental verification is required, such studies can facilitate the prioritization of novel therapeutic targets in the drug development process. Additionally, the structure of essential bacterial proteins identified via in silico strategies may be targeted using peptide-based drugs, which typically have high specificity and low toxicity [72]. In one study, researchers developed an ‘antibiotic peptide’ that targeted and disrupted the folded structure of gonococcal methionine aminopeptidase, an essential protein required for protein maturation [73]. This potential structure-disrupting peptide antibiotic was shown to inhibit gonococcal growth and infections in a human cervical epithelial cell model. 

Due to the reduction in treatment options for gonorrhea, alternative antimicrobial therapies are urgently needed to address the widespread dissemination of multidrug-resistant *N. gonorrhoeae*. To date, the application of nanotechnology and nanomaterials has received very little attention as a potential clinical intervention for gonococcal infection. In the first reported study investigating the antibacterial activities of nanomaterials against *N. gonorrhoeae*, Li et al. [74] found that silver nanoparticles (Ag NPs, 120 nm in diameter) exhibited the most potent antimicrobial activity against an ATCC strain of *N. gonorrhoeae*, characterized by reduced colony formation, damaged cell membranes, and decreased gonococcal viability by approximately 35% compared to the control group [74]. Furthermore, the 120 nm Ag NPs were not cytotoxic to human epithelial and fibroblast cell lines. Most significant, the combination of Ag NPs (120 nm) and cefmetazole (ineffective by itself) appeared to exert an ‘additive’ antimicrobial effect against clinical multidrug-resistant isolates of *N. gonorrhoeae*, with cefmetazole MICs for all tested multidrug-resistant isolates declining to below the sensitivity threshold when this antibiotic was combined with Ag NPs [74]. Additional research is clearly needed to determine whether Ag NPs act by disrupting cell membrane integrity and permeability and/or by complexing with cefmetazole to deliver a greater antibiotic dose to the pathogen. The toxicity of Ag NPs also needs to be assessed carefully. Nonetheless, the possibility that Ag NPs or other nanomaterials could be used as an adjuvant to bolster the anti-gonococcal activity of currently ineffective antibiotics, such as cefmetazole, against multidrug-resistant isolates is intriguing and should be explored further. Other investigations have focused on the selective delivery and stability of broad-spectrum silver-based microbicides in seminal fluid and their bactericidal activity against *N. gonorrhoeae* [75]. 

## 4. Discussion

Gonorrhea remains a challenging disease with wide-ranging clinical and social effects. For over 80 years, treatment of gonorrhea has evolved through changing antibiotic regimens as a result of *N. gonorrhoeae* resistance. Antibiotics such as penicillin, tetracyclines, quinolones, and macrolides, which previously were effective against *N. gonorrhoeae*, are no longer recommended as the first-line monotherapy due to widespread resistance among gonococci. Resistance to current extended-spectrum cephalosporin-based treatments has already emerged [76,77]. While antibiotic combinations will continue to offer limited options for gonorrhea treatment, there are currently no pipeline drugs in development to control multidrug-resistant *N. gonorrhoeae* infections. Although research may yield new therapeutic options, the genetic plasticity of the pathogen, coupled with its proficiency at acquiring genes encoding resistance determinants from the environment, will likely make the effectiveness of any new antibiotic short-lived.

It is therefore important that any long-term strategy designed to address the global burden of gonorrhea include vaccination. In a model-based epidemiological simulation study of vaccine efficacy, it was estimated that a 90% reduction in gonorrhea prevalence could be achieved in 20 years if a non-waning gonococcal vaccine of 50% effectiveness (or a 100% efficacy vaccine that wanes after 7.5 years) is administered to all 13 year olds [78]. There is currently no approved vaccine for protection against gonorrhea, and no candidate vaccine targets are at any advanced stage of clinical development. Potential gonorrhea vaccines have not progressed beyond the stages of discovery and preclinical evaluation to identify immune correlates and understand surrogates of protection. While there is a preponderance of scientific publications on gonorrhea, there is not much on vaccines. This can be attributed to historical failures at developing gonococcal vaccines.

A number of major challenges have been identified as limiting the successful development of vaccines against gonorrhea. One primary obstacle is that the infective mechanism and mode of drug resistance for this pathogen are not completely understood. Similarly, gonococcal mechanisms of host immune protection have not been clearly elucidated. Another challenge is that natural infection of *N. gonorrhoeae* does not confer immunity, nor does it protect against reinfection. Due to the ability of *N. gonorrhoeae* to suppress and divert host immune responses, only a transient and weak mucosal immune response is associated with gonorrheal infections. Most vaccine development is based on the principle of replicating the immunity of natural infection. Thus, conventional approaches such as the use of live-attenuated or inactivated *N. gonorrhoeae* will not be effective for gonococcal vaccination. Despite these limitations, the successful development of vaccines against human papilloma virus (HPV, the most common sexually transmitted infection), the development of vaccine technologies that employ antigen engineering, immune response optimization efforts, and enhanced targeted vaccine delivery have engendered optimism that development of a vaccine against *N. gonorrhoeae* is possible. Using the protein-based meningococcal vaccine as a prototype, for example, most vaccine optimists believe that an effective gonococcal vaccine can be developed. Other challenges to vaccine development include the observation that several strains of infective *N. gonorrhoeae* possess notable differences in their pathogenic behavior [24]. This heterogeneity implies the possibility of variation in the immune response depending on the strain and its associated virulence. Finally, perhaps the most relevant challenge to clinical vaccine development is the fact that *N. gonorrhoeae* is an obligate human pathogen, with the consequent absence of reliable test models for infection studies. The human-specific pathogenicity of *N. gonorrhoeae* renders typical animal models as poor estimations at best. Although animal models are still useful, they must be complemented with studies in human cells or clinical infections in order to produce meaningful data. Thus far, only female mice treated with 17-β estradiol have been reliably used as a small-animal model of lower genital tract infection [79,80]. Studies in this mice model have shown major reduction in the load and duration of gonococcal colonization with intraperitoneal administration of a multiantigenic peptide (MAP1) (a peptide mimic and an immunologic surrogate of the 2C7-OS epitope) [65], subcutaneously administered (and footpad-boosted) recombinant refolded porin delivered by virus replication particles (rrPorB-VRP) [81], and the nasally administered outer membrane vesicle (OMV) vaccine [82].

In the past 50 years, only three candidate gonococcal vaccines have progressed to clinical trials, with none offering reliable protection [29,30,31]. The experience and lessons learned from these trials suggest that a successful vaccine will need to be multivariate—capable of targeting multiple conserved epitopes. Moving forward, both new therapeutics and vaccines will need to be developed to keep pace with an evolving pathogen. There are only limited potential drug targets in *N. gonorrhoeae*, and the rate at which gonococci acquire resistance to existing drugs is cause for concern. Focusing on the non-variable host cell structures contacted by gonococci during adherence, colonization, and invasion may be a plausible approach for the development of new therapeutics. The success of maraviroc [83], an anti-attachment retroviral drug that targets host immune cells, may be a template in this regard. Other therapeutic options, including immunomodulators and monoclonal antibodies to enhance host immunity, may also be avenues of exploration for drug discovery scientists.
